# A Novel Proposal for Deep Learning-Based Diabetes Prediction: Converting Clinical Data to Image Data

**DOI:** 10.3390/diagnostics13040796

**Published:** 2023-02-20

**Authors:** Muhammet Fatih Aslan, Kadir Sabanci

**Affiliations:** Department of Electrical and Electronics Engineering, Karamanoglu Mehmetbey University, Karaman 70100, Turkey

**Keywords:** convolutional neural network, diabetes prediction, numeric-to-image, PIMA dataset, support vector machines

## Abstract

Diabetes, one of the most common diseases worldwide, has become an increasingly global threat to humans in recent years. However, early detection of diabetes greatly inhibits the progression of the disease. This study proposes a new method based on deep learning for the early detection of diabetes. Like many other medical data, the PIMA dataset used in the study contains only numerical values. In this sense, the application of popular convolutional neural network (CNN) models to such data are limited. This study converts numerical data into images based on the feature importance to use the robust representation of CNN models in early diabetes diagnosis. Three different classification strategies are then applied to the resulting diabetes image data. In the first, diabetes images are fed into the ResNet18 and ResNet50 CNN models. In the second, deep features of the ResNet models are fused and classified with support vector machines (SVM). In the last approach, the selected fusion features are classified by SVM. The results demonstrate the robustness of diabetes images in the early diagnosis of diabetes.

## 1. Introduction

The most prevalent chronic non-communicable disease in the world is diabetes, also known as diabetes mellitus. Diabetes is fatal or drastically lowers quality of life and affects more women than men [1]. Diabetes is particularly risky for pregnant women, and unborn children are likely to be affected by this disease. Generally, if the glucose level in the blood rises above the normal value, the person is considered diabetic. This is due to the inability of the pancreas in the human body to fully perform its task. The person’s blood sugar rises if the pancreas cannot utilize the insulin it produces or does not create enough of it. Diabetes can cause long-term damage to different organs such as the eyes, heart, kidneys and blood vessels [2]. There are three different types of diabetes: type 1, type 2 and gestational. In type 1 diabetes, the pancreas produces little or no insulin. Insulin therapy is needed. It is usually seen in young individuals (age < 30) or children. Type 2 is usually caused by insulin resistance and is more common in older (age > 65) and obese patients [3,4,5]. Gestational diabetes is hyperglycemia that occurs during pregnancy. In addition, after pregnancy, the risk of type 2 diabetes is higher in women, and in this case, babies are also at risk [6,7].

It is known that diabetes is a public health problem that affects 60% of the world’s population [8]. Although the main cause of diabetes is unknown, scientists think it is related to genetic factors and environmental conditions. There are currently 425 million diabetics worldwide, according to the International Diabetes Federation, and 625 million will develop the disease in the next 23 years [9,10]. It is essential to identify the disease at an early stage in order to stop this rise. Only early detection can stop the growth of the disease because there is no cure for diabetes, which is a lifetime condition. With the right treatment, regular nutrition and drugs, the disease can be managed after early diagnosis. [11,12]. However, a delayed diagnosis might result in heart conditions and serious harm to many organs. For the early diagnosis of diabetes, clinical (plasma glucose concentration, serum insulin, etc.) and physical data (for example, body mass index (BMI), age) are often used [13]. According to these data, a doctor carries out the diagnosis of the disease. However, making a medical diagnosis is a very difficult task for the doctor and can take a very long time. In addition, the decisions made by the doctor may be erroneous and biased. For this reason, the fields called data mining and machine learning are frequently used as a decision support mechanism for the rapid and accurate detection of diseases according to data [11,14,15].

Recent advances in computer technologies have led to the emergence of algorithms that allow human tasks to be performed faster and more automatically by computers. Tools such as data mining, machine learning and deep learning, which are generally referred to as artificial intelligence, have shown remarkable performance in interpreting existing data. Especially in the medical field, artificial-intelligence-based methods are used in the diagnosis or treatment of many different diseases as they provide fast and powerful results. Examples of these are diagnostic studies of cancer [16], diabetes [17], COVID-19 [18], heart diseases [19], brain tumors [20], Alzheimer’s [21], etc. For more comprehensive information on the applications of artificial intelligence in the medical field, research studies by Kaur et al. [22] and Mirbabaie et al. [23] can be reviewed. Artificial intelligence is very useful for the medical field. Thanks to the superior success of artificial intelligence in medical studies so far, it has recently become common to record medical big data in hospitals. Considering that each patient is a real data point, much numerical data such as electrocardiograms (ECG), electromyograms (EMG), clinical data, blood values or a large number of image data such as X-ray, magnetic resonance imaging (MRI) or computed tomography (CT) can be produced after medical records. In this sense, such medical records constitute an important part of big data in the medical field [24].

Machine learning algorithms are generally used to interpret (regression, classification or clustering) big data based on artificial intelligence. Thanks to these algorithms, the relationship between them is learned based on samples and observations of the data. Machine learning methods that are frequently used in this sense are artificial neural networks (ANN), support vector machines (SVM), k-nearest neighbors (k-NN), decision trees (DT) and naïve Bayes (NB). These methods directly learn the correlation between input and target data. However, with the developments in artificial intelligence and computer processors in the last decade, ANN has been further deepened, and deep learning, which applies both feature extraction and classification together, has come to the fore. Especially in big data applications, deep learning has given a great advantage over traditional machine learning methods [25]. The most frequently used model in deep-learning-based medical diagnosis/detection applications is convolutional neural network (CNN). CNN models are very popular due to both their deep architecture and high-level feature representation. Since the architecture designed for CNN is end to end, raw data are given as input and classes are obtained as output. Therefore, the designed architecture is very important for the performance of the CNN model [26]. Recently, however, researchers have adopted transfer learning applications and used popular CNN architectures such as ResNet [27], GoogleNet [28], Inception [29], Xception [30], VGGNet [31], etc. In different data-driven studies [32], the direct use of pre-trained or pre-designed CNN architectures has provided advantages in terms of both performance and convenience

### 1.1. Previous Artificial Intelligence Based Studies on Diabetes Prediction

This study performs deep-learning-based diabetes prediction using the PIMA dataset. In general, studies developed for diabetes prediction are based on machine learning or deep learning.

Some of the studies that applied diabetes prediction to the PIMA dataset using machine learning methods are as follows. Zolfaghari [33] performed diabetes detection based on an ensemble of SVM and feedforward neural network. For this, the results obtained from the individual classifiers were combined using the majority voting technique. The ensemble approach provided a better result than the individual classifiers with 88.04% success. Sneha and Gangil [34] performed diabetes prediction using many machine learning methods such as naïve Bayes (NB), SVM and logistic regression. The best accuracy was obtained with SVM with 77.37%. In addition, the authors applied feature selection for the PIMA dataset. The features with low correlation were removed. Edeh et al. [35] compared four machine learning algorithms, Bayes, decision tree (DT), SVM and random forest (RF), on two different datasets for diabetes prediction. In the experimental results with PIMA, the highest accuracy was obtained with SVM at 83.1%. Chen et al. [36] reorganized the PIMA data with preprocessing and removed the misclassified data with the k-means algorithm (data reduction). They then classified the reduced data with DT. As a result of the study, diabetes was predicted with an accuracy of 90.04%. Dadgar and Kaardaan [37] proposed a hybrid technique for diabetes prediction. First, feature selection was performed with the UTA algorithm. Then, the selected features were given to the two-layer neural network (NN) whose weights were updated by genetic algorithm (GA). As a result, diabetes estimation was provided with an accuracy of 87.46%. Zou et al. [38] used DT, RF, and NN models for diabetes prediction. They also used principal component analysis (PCA) and minimum redundancy maximum relevance (mRMR) to reduce dimensionality. As a result, RF performed more successful predictions than the others, with 77.21% accuracy. For other proposed studies based on machine learning, studies by Choudhury and Gupta [39] and Rajeswari and Prabhu [40] can be examined.

The following are some studies that use the PIMA dataset with deep learning models: For diabetes prediction, Ashiquzzaman et al. [41] created a network with an input layer, fully connected layers, dropouts and an output layer architecture. It fed the PIMA dataset features directly into this designed MLP and achieved an accuracy of 88.41% at the end of the application. Massaro et al. [42] created artificial records and classified these data with long short-term memory (LSTM) (LSTM-AR). The LSTM-AR classification result, which was stated as 89%, was superior to both LSTM and the multi-layer perceptron (MLP) with cross validation previously performed. Kannadasan et al. [43] designed a deep neural network that extracts features with stacked autoencoders and performs diabetes classification with softmax. The designed deep architecture provided 86.26% accuracy. Rahman et al. [44] presented a model based on convolutional LSTM (Conv-LSTM). They also experimented with traditional LSTM and CNN to compare the results. They applied grid search algorithm for hyperparameter optimization in deep models. For all models, the input layer was one dimensional (1D). After training and test separation, Conv-LSTM for test data outperformed other models, with 91.38% accuracy. Alex et al. [45] designed a 1D CNN architecture for diabetes prediction. However, missing values were corrected by outlier detection. Then, they preprocessed the data with synthetic minority oversampling technique (SMOTE), and the imbalance in the data were removed. They then fed the processed data into the 1D CNN architecture and achieved 86.29% accuracy. For other applications based on deep learning for diabetes prediction, the studies presented by Zhu et al. [46] and Fregoso-Aparicio et al. [47] can be examined.

Previous studies show that the PIMA dataset is often used for machine learning, 1D-CNN and LSTM structures. The numerical nature of the PIMA dataset has limited the feature extraction and classification algorithms that researchers can use. In this study, this limitation is overcome by converting numerical data to images. Thus, the PIMA numerical dataset will be applicable with popular CNN models such as ResNet, VGGNet and GoogleNet.

### 1.2. The Structure, Purpose, Differences and Contribution of the Study

Examining the previous studies mentioned in Section 1.1. reveals that various machine learning and deep-learning-based applications predict diabetes quite successfully for the PIMA dataset containing clinical data records. Similar to the PIMA dataset, many clinical data in the medical field are composed of numerical values. Using numerical values directly with conventional machine learning techniques is more typical because studies involving machine learning models such as SVM, NB, RF, DT, etc. feed raw data or data with small preprocessing directly to the model and give target (0 (negative)–1 (positive)) values to the output. Studies that design deep architecture using the same data feed the PIMA features either to the 1D convolution layer or to the fully connected layers. The study by Massaro, Maritati, Giannone, Convertini and Galiano [42] processed the PIMA dataset containing 1D data with a recurrent-neural-network (RNN)-based LSTM. Nevertheless, LSTM was designed for sequential data, whereas the PIMA dataset contains independent data.

Traditional machine learning techniques have been surpassed in many respects by deep learning, which has become more popular in recent years [48,49]. With the high-level capabilities they offer, particularly deep CNN models, they have shown greater performance, notably in computer vision applications. However, the PIMA dataset’s inclusion of numeric values has thus far prompted researchers to create 1D CNN models. Popular CNN models are created for computer vision, and therefore the input layer only accepts 2D data. These models are employed in transfer learning applications. As a result, feature extraction using well-known CNN models and a diabetes prediction using these models have not yet been established from this PIMA dataset containing independent numerical data. Therefore, in order to provide more successful diagnoses, transformation can be applied to the raw data in accordance with popular CNN models.

This study converts each sample in the PIMA dataset to images (diabetes images) to overcome this limitation. Each diabetes image has cells representing features in the PIMA dataset. The ReliefF feature selection algorithm [50,51,52] was also used to make the feature with high correlation more dominant in the image. After each feature is placed on the image according to its importance, data augmentation is applied for these images. In fact, the easy application of data augmentation for diabetes data is one of the important contributions of this study because compared to numerical data, data augmentation for images is an easier and more common technique. The augmented image data are then fed to the ResNet18 and ResNet50 CNN models and diabetes prediction is performed. In order to improve these current results, the features of both models are then fused and classified with SVM (CNN-SVM). Finally, feature selection is made with the ReliefF algorithm, among many fusion features, and these selected features are classified by SVM. At the end of the study, all these results are compared. According to the results, the CNN-SVM structure with selected fusion features provides more successful diabetes prediction than others. In addition, the results of the proposed method are compared with those of previous studies, and the method is proven to be effective. The contributions of the proposed method can be stated as follows:

- An application with an end-to-end structure is suggested for diabetes prediction.
- PIMA dataset with numeric values is converted to images.
- It is provided to use numerical diabetes data together with popular CNN models.
- During the conversion to the image, the importance of the features is taken into account.
- The proposed method is superior to most previous studies.

## 2. PIMA Indians Diabetes Dataset

In this study, the PIMA Indians Diabetes dataset, which is taken from the Kaggle data repository and is frequently preferred for diabetes prediction, is used. The access link is https://data.world/data-society/pima-indians-diabetes-database (Access Date: 8 June 2022). The National Institute of Diabetes and Digestive and Kidney Diseases provided the source data for this dataset. The dataset’s goal is to diagnose whether or not a patient has diabetes based on certain diagnostic metrics provided in the collection. All patients here, in particular, are PIMA Indian women over the age of 21.

The dataset includes the following measurements and ranges of clinical and physical characteristics. Pregnancies (number, [0–17]), glucose (value, [0–199]), blood pressure (mm Hg, [0–122]), skin thickness (mm, [0–99]), insulin (mu U/mL, [0–846]), BMI (kg/m^2^, [0–67.1]), diabetes pedigree function (PDF) (value, [0.078–2.42]), age (years, [21–81]), and outcome (Boolean- 0, 1). The data are entirely numerical and comprise a total of 8 features and 768 samples. Table 1 shows a few samples from the dataset.

## 3. Methodology

**Figure 1:**
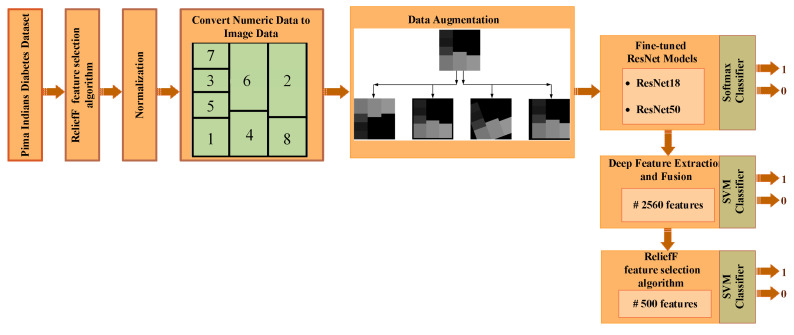
Application steps of proposed methods.

The methods used to determine the diabetes status of patients will be outlined in detail in this section. The steps of the proposed method are shown in Figure 1. The feature selection method initially selects the most useful features from the numerical data, as shown in Figure 1. The boundaries of all features are then adjusted for the numeric-to-image conversion stage once the numerical data has been normalized. The numerical to image conversion process is applied in such a way that the most effective features determined by the feature selection algorithm are dominant. The classification success of deep ResNet models is then increased by the use of data augmentation techniques. The three ResNet-based approaches suggested in this study are used to classify data in the final stage. Below, we go over each of these processes in more detail.

### 3.1. ReliefF Feature Selection Algorithm

To improve classification capability, a variety of feature reduction strategies have been explored in the literature [53]. In the literature, ReliefF is one of the distance-based feature selectors. ReliefF, developed by Kira and Rendell [52] in 1992, is one of the most successful feature filtering methods.

Dimension reduction strategies aid in the removal of superfluous attributes from a data set. These technologies aid in data compression, which saves storage space. It also shortens the time required for computational complexity and reduces the amount of time it takes to attain the same goal [54].

Kononenko [55] improved the algorithm for multi-class issues in 1994. With the help of this algorithm, feature selection can be performed successfully. The ReliefF algorithm is highly efficient and does not impose any restrictions on the data kinds’ features. The ReliefF method assists in the solution of many classes of issues by selecting the nearest neighboring samples from each sample in each category [56].

ReliefF seeks to expose the connections and consistency found in the dataset’s properties. Furthermore, by constructing a model that addresses the proximity to samples of the same class and distance to samples of different classes, it is feasible to discover the significant features in the dataset. Between samples of distinct qualities, the ReliefF model chooses neighboring attributes that are closest to each other [54]. The dataset is divided into two components in this model: training and test data. $R_{i}$ random samples are chosen from the training set, and the difference function diff is used to calculate the nearest neighbors of the same and different classes to identify the nearest neighbors to the selected $R_{i}$ sample, as illustrated in Equation (1). When identifying nearest neighbors, the diff function is also utilized to compute the distance between instances. The total distance is simply the sum of all attribute differences (i.e., Manhattan distance) [51].

Equation (1) is used to determine the difference between two separate $I_{1}$ and $I_{2}$ samples for the attribute A and to discover the closest distance between samples. The nearest neighbor H from the same class and the nearest neighbor M from a different class are chosen. The distance of adjacent sample $A_{f}$ in the class and between the classes is compared based on the values of $R_{i}$, M, H, and the dataset’s weighting vector. The $WA_{f}$ weight is calculated as a result of the comparison by giving less weight to the distant attributes [57]. These processes are performed m times for each attribute, and the weight values are calculated for each attribute. The weights are updated using Equation (2) [55,58].

(1) $$diff\left(A,I_{1},I_{2}\right)=\frac{\left|value\left(A,I_{1}\right)-value\left(A,I_{2}\right)\right|}{\max\left(A\right)-\min\left(A\right)}$$

(2) $$W_{new}\left(A_{f}\right)=W_{old}\left(A_{f}\right)+\frac{diff\left(A_{f},R_{i},M\right)}{m}-\frac{diff\left(A_{f},R_{i},H\right)}{m}$$

**Figure 2:**
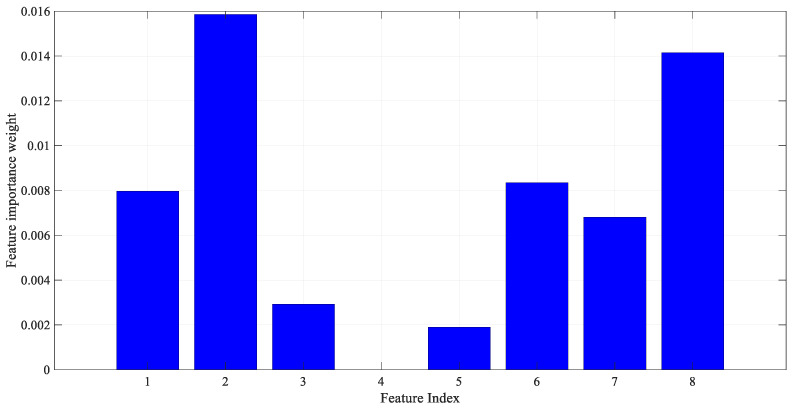
Importance weight of features in the PIMA dataset.

As a result of applying the ReliefF feature selection method described above to the PIMA dataset features, the importance weight of each feature is shown in Figure 2. The number of nearest neighbors was also determined as 10. As seen in Figure 2, the most effective features from the PIMA numerical data were determined by the ReliefF algorithm.

### 3.2. Normalization of Data

In artificial intelligence studies, normalizing data containing many features is a known process. Because different features have different limits. Setting features to the same or similar range, i.e., normalization, improves learning performance. The PIMA dataset also has different lower and upper bound values, as seen in Table 1. In this sense, normalization of these values is necessary. In addition, normalization is vital for the numeric-to-image conversion process in the proposed implementation because the value of each feature must be located on the image that represents that sample. According to the amplitude of the feature, the cell in the corresponding image has a brighter color. Therefore, the maximum and minimum values for all features must be the same.

**Figure 3:**
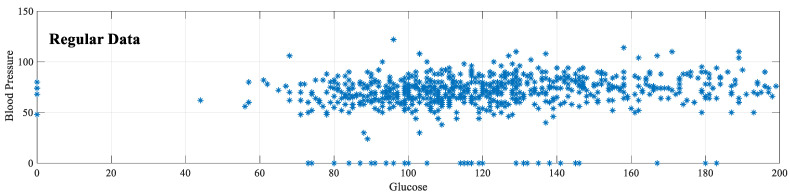

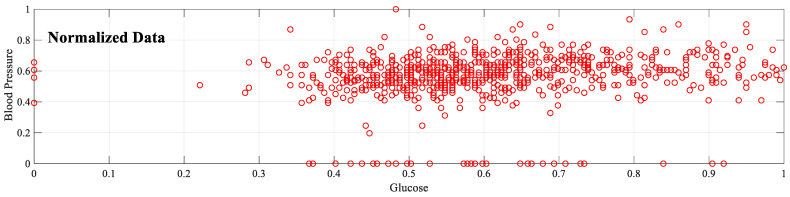
Min–max normalization of PIMA dataset.

The preferred method for normalization is feature scaling. With this method, feature values are rescaled to a certain range. The feature scaling method used in this study is the min–max normalization method. In this method, the new sample value ($\hat{x}$) is determined according to the maximum ($x_{max}$) and minimum ($x_{min}$) values of the features. As a result of normalization, all features are distributed between 0–1. In the application phase, normalization is applied for eight features in the PIMA dataset. Figure 3 shows that after this normalization, the glucose [0–199] and blood pressure [0–122] values range from 0–1. Equation (3) shows the formula for the min–max normalization method.

(3) $$\hat{x}=\frac{x-x_{min}}{x_{max}-x_{min}}$$

### 3.3. Conversion of Numeric Data to Image Data

Although the number of image data in the medical field has increased considerably recently, there is still a large amount of numerical data available. Although numerical values are easily and cheaply obtained, the interpretation of these data is usually performed by machine learning methods. Recently proposed deep architecture studies prefer 1D CNN structures that take these numerical values as input because popular CNN models, which provide significant improvements in computer vision, cannot be used directly for such data. Because for these models, 2D data should be given as input to the input layer. CNN models such as ResNet, VGGNet, GoogleNet, etc., have an architecture designed for image data. Therefore, the inability to analyze datasets containing 1D samples with these powerful models is a major disadvantage in terms of both application diversity and prediction performance. This section discusses the conversion of numeric data to images to overcome this limitation in the PIMA dataset, which is a numeric dataset.

**Figure 4:**
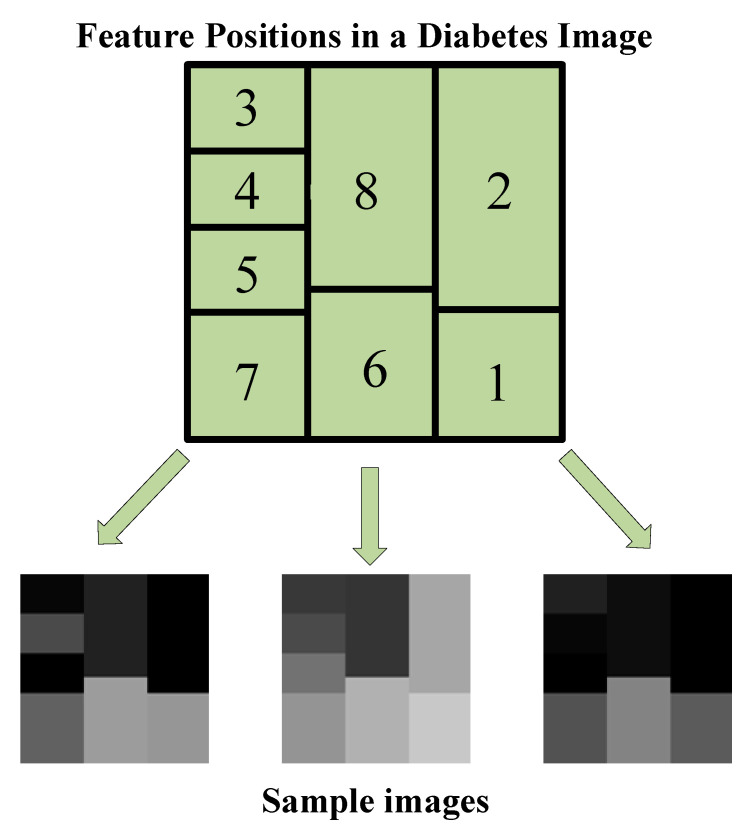
Conversion selected features to image (numeric to image).

In the process of converting PIMA data to images, the principle of determining the brightness of a specific region (cell) in the image according to the amplitude of each feature is adopted. In fact, each feature can be viewed as a piece of the sample image’s puzzle. For each sample in the PIMA dataset, the 120 × 120 image structure shown in Figure 4 is used. The index on each cell corresponds to the feature index in the PIMA dataset. That is, Figure 4 shows feature locations in a sample image. In Figure 4, the location and size of features are determined not randomly but based on feature importance. As seen in Figure 2 as a result of the ReliefF algorithm, the order of importance of features is 2-8-6-1-7-3-5-4. Therefore, a larger cell is assigned for the more important feature. Each cell is colored according to the amplitude of the corresponding feature value. Because all data were previously normalized, each feature value ranges from 0 to 1. Each feature value is multiplied by 255, resulting in images with cells with brightness values between 0 and 255. Therefore, the resulting images are in gray spaces. Some sample diabetes images are shown in Figure 4.

As a result of applying the aforementioned image conversion method on the PIMA dataset, one image for each sample (that is, 768 images in total) is formed. These images, with all features included, can now be used in CNN models that require 2D data input. Furthermore, image data augmentation methods are easily applicable to these image data. For this purpose, the image structure in Figure 4 is designed asymmetrically because in the data augmentation stage, all images must be reproduced differently from each other.

### 3.4. Data Augmentaion

The number of samples directly influences the success of deep learning approaches. However, accessing a significant volume of data is not always possible. As a result, researchers artificially increase the size of a training dataset by producing modified versions of the images in the dataset. These techniques, which are applied to raw images for this purpose, are known as data augmentation techniques.

**Figure 5:**
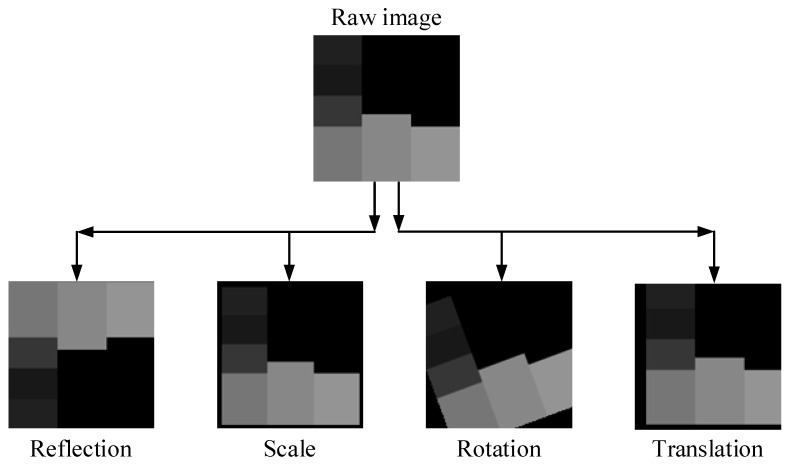
Data augmentation methodologies and sample augmented images.

In this study, the diabetic data contains 768 numerical samples in total, and hence 768 images are created during the conversion from numerical to image data. Data augmentation techniques are used because this amount is insufficient for a deep learning implementation. To ensure data diversity and robust training, four different data augmentation techniques (rotation, scale, reflection and translation) are applied to all images produced, as in Figure 4. Table 2 shows the lower and upper limit values for these data augmentation techniques. Additionally, Figure 5 shows the new diabetes images produced as a result of data augmentation techniques.

After data augmentation, each original diabetes image is reproduced in four different ways. As a result, a total of five artificial samples is obtained from one sample. The sample numbers of the classes before and after the data augmentation stage are shown in Table 3. As a result of the data augmentation, the total number of images reached 3840.

### 3.5. Diabetes Prediction via ResNet Models

After data augmentation, images separated into 80% training and 20% testing are fed to the CNN model. In this study, diabetes estimation is provided with the ResNet18 and ResNet50 models, which are frequently used for comparison purposes. Many studies apply ResNet models widely because of the advantages they provide [59]. What makes ResNet preferable is that it transmits residual values to the next layers to avoid the vanishing gradients problem, for which it uses residual blocks. There are ResNet models with different depths. The depths of the ResNet18 and ResNet50 models used in this study are 18 and 50, respectively.

**Figure 6:**
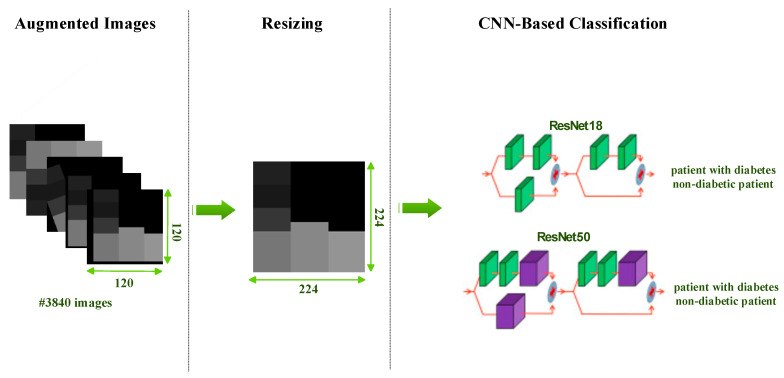
Classification of diabetes images as diabetic (1) and nondiabetic (0) with ResNet models.

This study performs diabetes detection with existing models instead of designing a new CNN architecture. With only minor modifications (fine-tuning), existing ResNet models are adapted to our work. For both models, the last two layers of existing models are removed and replaced with two fully connected output layers and a classification (softmax) layer. In addition, while the diabetes images produced are 120 × 120, the input size for ResNet models should be 224 × 224. Therefore, all diabetes images are resized before and during training (see Figure 6). Information about the results obtained after the training and testing phases will be discussed in the results section.

### 3.6. Deep Feature Extraction, Feature Selection and Classification

**Figure 7:**
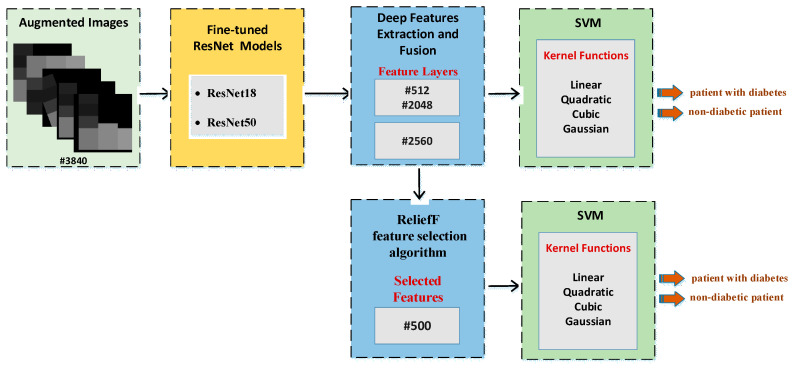
Implementation steps of the proposed CNN-SVM approach.

While the previous section directly uses fine-tuning of ResNet models, this section describes the CNN-SVM structure. In other words, CNN is used for feature extraction and SVM is used for classification. This approach has been frequently preferred recently to increase the classification accuracy [60]. Two different experimental applications are presented at this stage. The features obtained with the CNN models in the previous stage are combined and fed to the SVM. In the previous step, 512 deep features were extracted from the ResNet18 model, and 2048 deep features were extracted from the ResNet50 model. Then, these features were combined to obtain a total of 2560 deep features, with 80% and 20% of these deep features being divided into two groups for training and testing. In the first experimental stage, these deep features are classified by the SVM machine learning algorithm. For this classification, linear, quadratic, cubic and Gaussian SVM kernel functions are used and results are obtained. In the second stage, the most effective 500 features from a total of 2560 features extracted from ResNet models are selected using the ReliefF feature selection algorithm. These 500 selected features are classified by the SVM machine learning algorithm. Similarly, at this stage, classification is made for SVM using linear, quadratic, cubic and Gaussian SVM kernel functions. All results are then compared. Figure 7 shows the proposed CNN-SVM structure.

**Figure 8:**
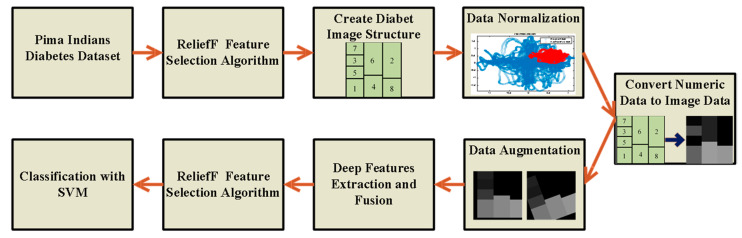
Application flow chart in the last step.

The results of the experimental studies are discussed in Section 4. Experimental application in the last step provided the most successful results. The flow graph containing the applications of this step is shown in Figure 8.

## 4. Results and Discussion

In this section, the results of the proposed approach are discussed. All deep learning applications for diabetes prediction were performed using a laptop with Intel Core i7-7700HG processor, NVIDIA GeForce GTX 1050 4 GB graphics card and 16 GB RAM. Applications are developed in Matlab environment. Figure 8 can be taken as reference for the software design or code implementation of the proposed approach. The algorithm of the method was created as in Figure 8. Toolbox and libraries used directly during coding prevented software complexity. Toolboxes used in this context are Machine Learning Toolbox, Deep Learning Toolbox and Image Processing Toolbox.

In order to demonstrate the superiority of the proposed method, results are produced with three different approaches. Methodological information about these three approaches has been shared in detail in the previous section. In the first approach, classification is performed with fine-tuned ResNet models to perform the diabetes prediction using diabetes images after data augmentation. For this, the ResNet18 and ResNet50 models are fine-tuned, and the output layer is changed according to the two classes. Then, 3840 diabetes images are divided into two groups as 80% and 20% training data and test data, respectively. While the models are trained using the training data, the performance of the network is obtained using the test data. In the second approach, deep features extracted from two fine-tuned ResNet models are combined and these fusion features (2560) are classified by the SVM machine learning method. The performance of SVM differs according to the kernel function used. Therefore, in the second approach, classification accuracies are obtained by using linear, quadratic, cubic and Gaussian kernel functions and compared with each other. In the last approach, namely the proposed method, the most important 500 features from a total of 2560 fusion features extracted from fine-tuned ResNet models are selected with the ReliefF feature selection algorithm. In this way, we aimed to achieve similar success with fewer features. These features are classified with SVM as in the second approach. Classification results are obtained with linear, quadratic, cubic and Gaussian kernel functions, and the results are compared with other approaches.

ResNet models are trained once for all the approaches mentioned above. In other words, as a result of the three approaches, the features obtained with the ResNet models are the same. The parameters used for training ResNet models are: Mini Batch Size: 32; Max Epochs: 5; Learn Rate Drop factor: 0.1; Learn Rate Drop period: 20; Initial Learn Rate: 0.001. In addition, the optimizer used to update the weights during the training process is Stochastic Gradient Descent with Momentum.

**Figure 9:**
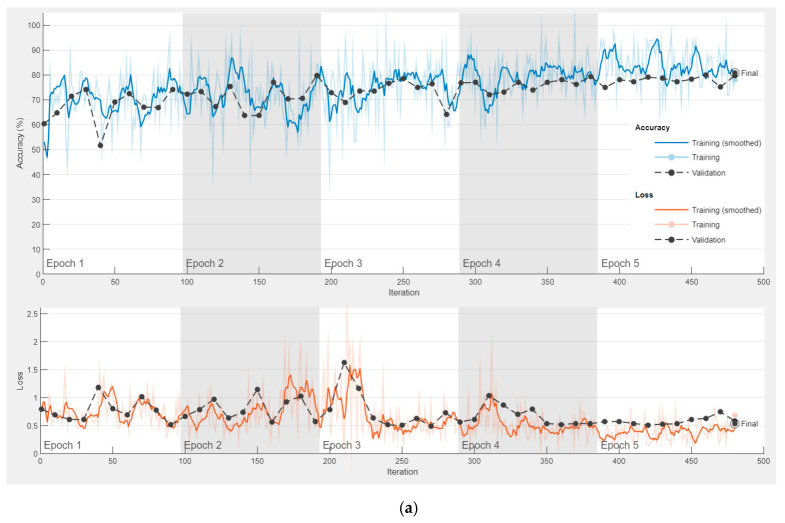

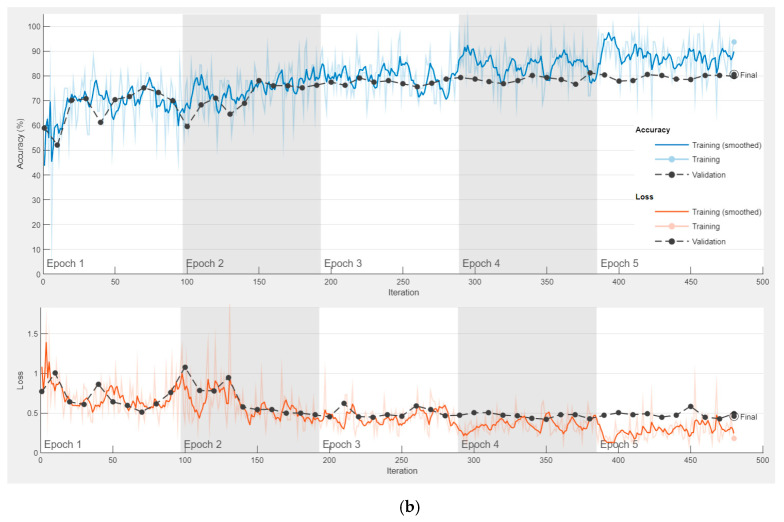
Training and loss graphics of ResNet models. (**a**) ResNet18. (**b**) ResNet50.

**Figure 10:**
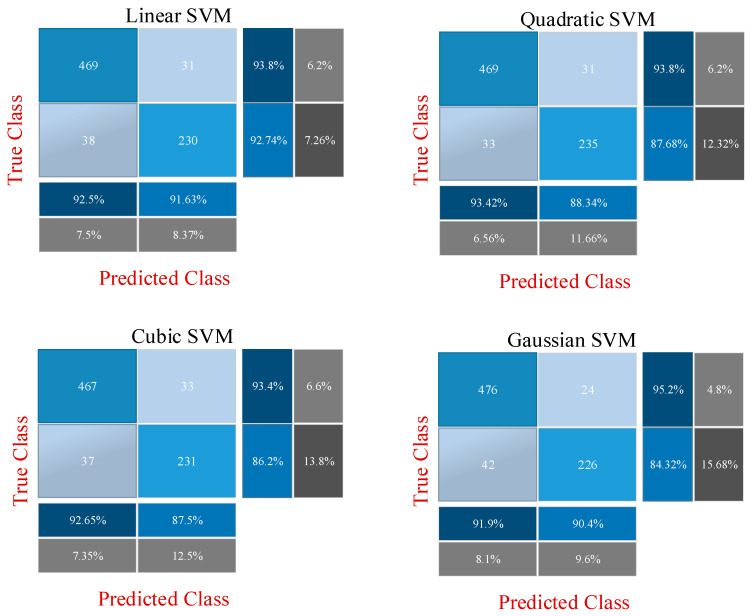
Confusion matrices obtained as a result of classification of all fused features with SVM.

**Figure 11:**
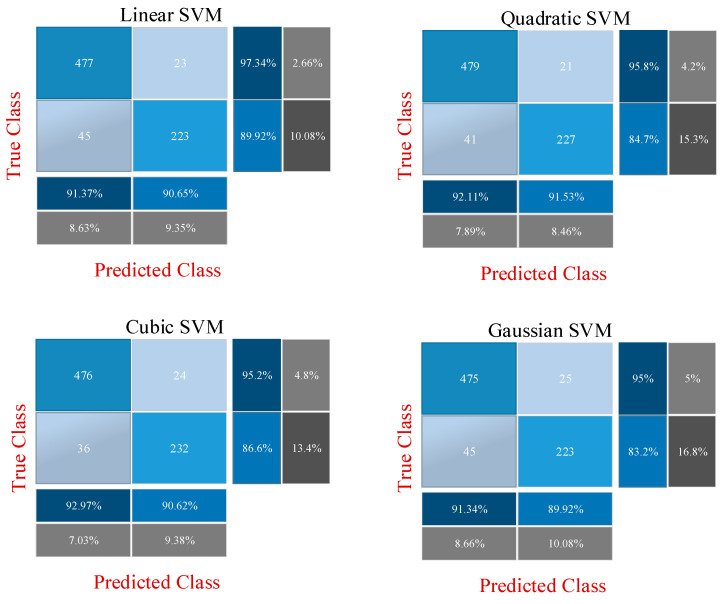
Confusion matrices obtained as a result of classification of selected features with SVM.

After training is performed in the first approach, the features obtained in the first approach are used in other approaches. The accuracy and loss graph of the first approach, obtained during the training and testing phase of the ResNet18 and ResNet50 models, is shown in Figure 9. It is clear that overfitting does not occur during the training phase. In the second and third approaches, the CNN model is not trained, and 512 and 2048 features are extracted from ResNet18 and ResNet50, respectively, through fully connected layers used directly. The confusion matrixes obtained as a result of the classification of these features with SVM are shown in Figure 10 and Figure 11. Figure 10 shows the application results obtained with the second approach using all the fusion features. Figure 11 shows the final application results that classify the selected features from the fusion features.

**Figure 12:**
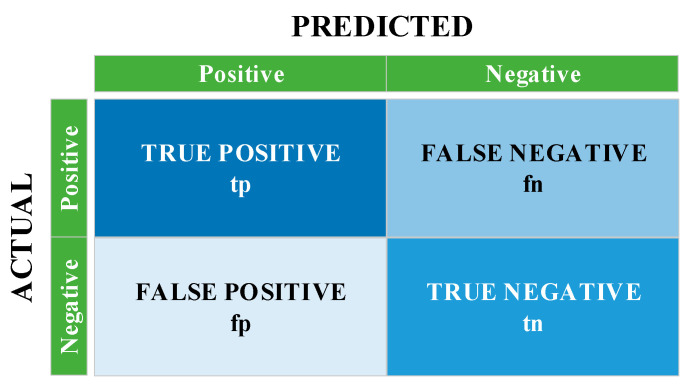
Structure of confusion matrices.

The confusion matrix structure that enables the calculation of these metrics is shown in Figure 12. The performance of the system is measured with the tp, tn, fp and fn values in this matrix. Using these values, accuracy, specificity, precision, sensitivity, F1-score and MCC performance metrics are calculated with the help of the formulas between Equations (4) and (9). Table 4 shows the performance metrics obtained as a result of the three approaches.

(4) $$Accuracy=\frac{tp+tn}{tp+fp+tn+fn}\times 100$$

(5) $$Specificity=\frac{tn}{tn+fp}$$

(6) $$Precision=\frac{tp}{tp+fp}$$

(7) $$Sensitivity=\frac{tp}{tp+fn}$$

(8) $$F1-score=\frac{2tp}{2tp+fp+fn}$$

(9) $$MCC=\frac{\left(tp\times tn\right)-\left(fn\times fp\right)}{\sqrt{\left(tp+fn\right)\times \left(tn+fp\right)\times \left(tp+fp\right)\times \left(tn+fn\right)}}$$

According to Table 4, the highest accuracy in the first approach is obtained with the fine-tuned ResNet18 model. The accuracy rates obtained with ResNet18 and Resnet50 are 80.86% and 80.47%, respectively. In the second approach, in the classification made with SVM using 2560 features, the highest accuracy is calculated as 91.67% with the quadratic kernel function. In the last approach, in the classification made with the 500 most effective features selected by the feature selection algorithm, the highest accuracy is calculated with the SVM/cubic kernel function of 92.19%. The results of the first approach showed that converting diabetes data from numeric to image is an effective technique because these images were successfully classified with ResNet models. The second approach shows that fusing the features of different CNN models highly affects the success. In addition, SVM also showed a successful classification performance. The last approach showed that higher achievement can be achieved with fewer features. The results obtained with the last approach are compared with previous studies using the PIMA dataset, as shown in Table 5. As can be seen, the method proposed in our study outperformed many previous studies. Considering the methodological knowledge of previous studies, the numerical nature of the PIMA dataset has led researchers to use algorithms fed with numerical data such as traditional machine learning, 1D-CNN and LSTM. This study, unlike previous studies, transformed the PIMA dataset into image data and thus made the PIMA dataset suitable for popular CNN models.

## 5. Conclusions, Discussion and Future Works

Diabetes is a chronic disease that limits people’s daily activities, reduces their quality of life and increases the risk of death. In the past, machine learning and DNN solutions have been developed using clinical data and various diabetes prediction studies have been carried out. Despite the encouraging results of these studies, the numerical nature of clinical registry data has limited the use of popular CNN models. In this study, popular CNN models were used to determine the diagnosis of diabetes. Since these CNN models require two-dimensional data input, numerical clinical patient data (PIMA dataset) were first converted to images in this study. In this way, each feature was included in the sample image. This process was not performed randomly, and the most effective feature was made to stand out more in the image. During this process, the ReliefF feature selection method was used to determine the most effective features. After the number of generated images was increased by data augmentation and their size was adjusted for the ResNet model, diabetes prediction was carried out with three different approaches.

Diabetes images were successfully classified with the first approach using the fine-tuned ResNet18 and ResNet50 models. In the second approach, SVM was used to classify a total of 2560 deep features extracted from the fully connected layers of both ResNet models. In the last approach, the most effective 500 of these deep features were selected using the ReliefF feature selection algorithm, and the selected features were classified by SVM. The most successful prediction was obtained with the third approach. The accuracy of the classification using the SVM/cubic model with 500 selected features was 92.19%. All these classifications were performed on the image data. The conversion to image data removed the algorithm limitation that can be used for the PIMA dataset. In this way, the PIMA dataset or similar numerical data can be analyzed with different CNN models capable of extracting high-level and complex features. An application containing image data can be analyzed more diversely and comprehensively than an application containing numerical data because the different artificial intelligence combinations that can be applied to the image data are very rich. The results obtained with the ResNet18 and ResNet50 models in this study, therefore, outperform previous studies. For example, the number and variety of features can be increased with different CNN models. Based on all this, the experimental results have shown that converting clinical data into images is an effective technique.

The method proposed in this study can also be applied for different numerical data. Deep-learning-based studies have reduced the dependency on features and the designed architecture has come to the fore. However, the method proposed in this study is valuable in that deep and comprehensive architectures can also be used for numerical data. This application may involve more processing steps than studies using raw data directly. However, the generation of image data paves the way for further improvement of diabetes prediction performance because CNN models in many different architectures are now applicable to numerical data. Moreover, data augmentation can now be easily applied to diabetes images. In addition, the application results show that the fusion features used in the CNN-SVM architecture greatly increase the success. Additionally, using selected features, CNN-SVM is less costly and provides more accurate predictions. Based on these situations, the important trend of experimental simulation studies can be explained as follows: selected fusion features increase the performance of the system, although they are fewer in number. In addition, the CNN-SVM structure is quite effective. Different applications with fewer, more effective and more diverse features increase the classification accuracy of the system.

In future studies, it is planned to use of different CNN models and feature selection methods to improve diabetes prediction performance. A greater variety of features will be obtained by using more CNN models. In this case, it is expected that the classification accuracy will increase. In addition, future studies plan to apply the produced diabetes images with transformer-based networks.

## Figures and Tables

**Table 1:** Some examples of Pima Indians Diabetes dataset.

Pregnancy [0–17]	Glucose [0–199]	Blood Pressure [0–122]	Skin Thickness [0–99]	Serum Insulin [0–846]	BMI [0–67.1]	PDF [0.078–2.42]	Age [21–81]	Outcome (0–1)
1	89	66	23	94	28.1	0.167	21	0
2	197	70	45	543	30.5	0.158	53	1
1	189	60	23	846	30.1	0.398	59	1
1	103	30	38	83	43.3	0.183	33	0
9	171	110	24	240	45.4	0.721	54	1
5	88	66	21	23	24.4	0.342	30	0
2	141	58	34	128	25.4	0.699	24	0
2	100	66	20	90	32.9	0.867	28	1
7	83	78	26	71	29.3	0.767	36	0
7	160	54	32	175	30.5	0.588	39	1

**Table 2:** Approaches to data augmentation’s lower and upper limitations.

Parameter Name	Lower Limit	Upper Limit
Reflection	-	-
Rotation	−30°	30°
Scale	0.9	1.1
Translation	−10	+10

**Table 3:** Examination of all data before and following data augmentation.

Class	0 (Negative)	1 (Positive)	Total
Before data augmentation	500	268	768
After data augmentation	2500	1340	3840

**Table 4:** Performance metrics for the three approaches.

Model	Kernel	Acc. (%)	Spec.	Prec.	Sens.	F1-Score	MCC
ResNet18	-	80.86	0.6689	0.8142	0.8947	0.8526	0.5868
ResNet50	-	80.47	0.5734	0.7826	0.9474	0.8571	0.5832
SVM with 2560 features	Linear	91.02	0.8582	0.9250	0.9380	0.9315	0.8012
	Quadratic	91.67	0.8769	0.9343	0.9380	0.9361	0.8163
	Cubic	90.89	0.8619	0.9266	0.9340	0.9340	0.7988
	Gaussian	91.41	0.8433	0.9189	0.9520	0.9352	0.8090
SVM with 500 selected features	Linear	91.15	0.8321	0.9138	0.9540	0.9335	0.8030
	Quadratic	91.93	0.8470	0.9212	0.9580	0.9392	0.8206
	**Cubic**	**92.19**	**0.8657**	**0.9297**	**0.9520**	**0.9407**	**0.8268**
	Gaussian	90.89	0.8321	0.9135	0.9500	0.9314	0.7972

**Table 5:** Comparative analysis with previous works.

Previous Work	Method	Accuracy (%)
Zolfaghari [33]	Ensemble of SVM and NN	88.04
Sneha and Gangil [34]	Feature Selection and SVM	77.37
Srivastava et al. [61]	ANN	92.00
Edeh, Khalaf, Tavera, Tayeb, Ghouali, Abdulsahib, Richard-Nnabu and Louni [35]	SVM	83.1
Massaro, Maritati, Giannone, Convertini and Galiano [42]	LSTM-AR	89
Dadgar and Kaardaan [37]	UTA-NN and GA	87.46
Zou, Qu, Luo, Yin, Ju and Tang [38]	mRMR-RF	77.21
Ashiquzzaman, Tushar, Islam, Shon, Im, Park, Lim and Kim [41]	Deep MLP	88.41
Kannadasan, Edla and Kuppili [43]	Stacked Autoencoders-DNN	86.26
Rahman, Islam, Mukti and Saha [44]	Conv-LSTM	91.38
Alex, Nayahi, Shine and Gopirekha [45]	DCNN/SMOTE/Outlier Detection	86.29
Kalagotla et al. [62]	Stacking of MLP, SVM, LR	78.2
Jakka and Vakula Rani [63]	LR	77.6
**Proposed method**	Diabetes images: ResNet18 and ResNet50-ReliefF	92.19

## Data Availability

The data utilized in this work can be found at https://data.world/data-society/pima-indians-diabetes-database (accessed on 8 June 2022).
